# 

**Published:** 1874-09

**Authors:** 


### CONTENTS:

ORIGINAL COMMUNICATIONS:

ART. I. On the Use of Ice in Painful Conditions of the Bladder and Rectum. By H. M. Lyman, M.D...... 513

ART. II. Multilocular Sero-Cystic Ovarian Tumor successfully treated by means of the Electropuncture. By P. S. Hayes, M.D.................. 517

ART. III. Case of Retention of Placenta. By C. E. Ristine, M.D...... 522

ART. IV. Case of Strangulated Hernia. By J. W. Robbins, M.D...... 524

ART. V. Fœtal Auscultation. By Albert B. Strong, M.D .............. 524

ART. VI. Record of Observations, etc., with a view of determining Sex in Utero. By D. A. K. Steele, M.D... 530

PROGRESS IN MEDICAL SCIENCES........ 533

REPORTS OF SOCIETIES ................. 544

THE HOSPITALS ...... ............... 557

EDITORS' BOOK TABLE .............. .. 563

EDITORIAL ............................ 567

MORTALITY STATISTICS ................. 574

